# Plasmalogens and Octanoylcarnitine Serve as Early Warnings for Central Retinal Artery Occlusion

**DOI:** 10.1007/s12035-024-04093-9

**Published:** 2024-03-08

**Authors:** Chuansen Wang, Ying Li, Jiaqing Feng, Hang Liu, Yuedan Wang, Yuwei Wan, Mengxue Zheng, Xuejie Li, Ting Chen, Xuan Xiao

**Affiliations:** 1 Department of Ophthalmology, Renmin Hospital of Wuhan University, No. 238 Jie Fang Road, Wuhan, 430060 Hubei China; 2 Department of Clinical Laboratory, Institute of Translational Medicine, Renmin Hospital of Wuhan University, Wuhan, China

**Keywords:** Central retinal artery occlusion, Metabolomics, Differential metabolites, Prediction model, Clinical parameter

## Abstract

Central retinal artery occlusion (CRAO) is a kind of ophthalmic emergency which may cause loss of functional visual acuity. However, the limited treatment options emphasize the significance of early disease prevention. Metabolomics has the potential to be a powerful tool for early identification of individuals at risk of CRAO. The aim of the study was to identify potential biomarkers for CRAO through a comprehensive analysis. We employed metabolomics analysis to compare venous blood samples from CRAO patients with cataract patients for the venous difference, as well as arterial and venous blood from CRAO patients for the arteriovenous difference. The analysis of metabolites showed that PC(P-18:0/22:6(4Z,7Z,10Z,13Z,16Z,19Z)), PC(P-18:0/20:4(5Z,8Z,11Z,14Z)) and octanoylcarnitine were strongly correlated with CRAO. We also used univariate logistic regression, random forest (RF), and support vector machine (SVM) to screen clinical parameters of patients and found that HDL-C and ApoA1 showed significant predictive efficacy in CRAO patients. We compared the predictive performance of the clinical parameter model with combined model. The prediction efficiency of the combined model was significantly better with area under the receiver operating characteristic curve (AUROC) of 0.815. Decision curve analysis (DCA) also exhibited a notably higher net benefit rate. These results underscored the potency of these three substances as robust predictors of CRAO occurrence.

## Introduction

Central retinal artery occlusion (CRAO) is an ophthalmological emergency which mainly refer that the interruption of blood flow of central retinal artery occlusion [1–3]. The outcome of CRAO is very serious, which may cause loss of functional visual acuity and damage retinal cells irreversibly within hours [4, 5]. In addition, there is an increased risk of stroke and myocardial ischemia after CRAO[6–8]. Therefore, early identification of possible CRAO patients and early intervention is extremely important to save vision of patient. A 10-year follow-up of the cohort showed that AV incision, arteriole wall opacity, and retinal vein obstruction predicted the occurrence of retinal embolism, but no subsequent studies supported this view [9]. Currently, studies of biomarkers for CRAO have focused on hematological markers such as Neutrophil/lymphocyte Ratio, but these markers are primarily used to assess a patient's systemic inflammatory status. And exhibits greater prognostic value in assessing the clinical outcomes of individuals with CRAO [10–12]. However, there is still no suitable biomarker to predict the occurrence of CRAO. New biomarkers and targets are needed for CRAO.

Metabolomics is mainly a discipline that studies the types, quantities, and their changes of metabolites (<1500 Da) caused by organisms caused by external stimuli, pathophysiological changes, and genetic mutations [13, 14]. Because metabolites can directly reflect the metabolic pathway at a certain moment, they have the potential to magnify even subtle changes at the gene or protein level [15, 16]. At present, metabolomics has shown a great role in the identification of biomarkers in the field of other cardiovascular and cerebrovascular diseases, such as myocardial infarction and stroke [17–20]. Given the similarities in pathogenesis between retinal artery occlusion and conditions like stroke and myocardial infarction, metabolomics may be helpful for the discovery of potential biomarkers for CRAO [21, 22]. Employing serum as sample may easily reflect systemic changes of human body, making it potentially valuable for uncovering the underlying mechanisms of CRAO, a type of vascular disorder. However, the metabolomics of CRAO based on serum is still an unexplored territory for researchers.

In this study, we conducted a comparison between the venous blood of CRAO patients and control patients utilizing non-targeted metabolomics. This approach allowed us to discern alterations in metabolite profiles among CRAO patients. Additionally, we compared arterial blood collecting from the embolization site in the anterior segment of CRAO patients before thrombolytic surgery and venous blood. This dual approach aimed to provide a more comprehensive understanding of the lesions and possible information about emboli associated with CRAO. Our aim is to investigate the potential underlying mechanisms of CRAO through the analysis of the metabolome and identifying potential biomarkers.

## Material and Methods

### Patient Cohort

This study was approved by the Ethical Committee Board of Renmin Hospital of Wuhan University and followed the principles of the Declaration of Helsinki. Written informed consent was obtained from all participants enrolled in this study. Inclusion criteria: (1) age 18–75 years; (2) CRAO patients were diagnosed with existing guidelines, including sudden vision loss, positive RAPD, retinal ischemic edema, and delayed arterial filling on angiography; (3) the patient or surrogate consent to take part in the study; (4) blood samples were collected within 72 h of symptom onset. Exclusion criteria include the following: (1) the patient had a history of CRAO; (2) the patient had no coronary artery disease, stroke, malignancy, severe renal insufficiency (eGFR<30 mL/min), liver disease, or lung disease.

Demographic, clinical, and laboratory data of patients were obtained, including factors such as age and gender. Venous blood samples were prospectively collected from patients after overnight fasting in the morning at first admission. Arterial blood samples were taken from upstream of the embolization site of CRAO near the embolus prior to interventional injection of thrombolytic agents. In total, venous blood samples were collected from 37 patients with CRAO, and arterial blood samples were collected from 28 of these patients. At the same time, we collected blood samples of 24 cataract patients as the control group.

Patients with hypertension were defined as those with blood pressure above 140/90 mmHg measured on two or more separate occasions or who had a hypertension history. Hematological indicators, including red blood cell count (RBC) and hemoglobin (Hb), were measured using XS-1000i fully automated hematology analyzer (Sysmex) and accompanying reagents. The lipid profiles, triglycerides (TG), total cholesterol (TCh), high-density lipoprotein cholesterol (HDL-C), low-density lipoprotein cholesterol (LDL-C), Lipoprotein A (LPA), Apolipoprotein A1 (ApoA1), Apolipoprotein B (ApoB) and Apolipoprotein A1b (ApoA1b), were measured by ADVIA 2400 biochemical analyzer (Siemens). Non-high-density lipoprotein cholesterol (Non-LDL-C) was calculated as TCh minus HDL-C.

The levels of prothrombin time (PT), thrombin time (TT), activated coagulation time (ACT), activated partial thromboplastin time (APTT), international normalized ratio (INR), fibrinogen (Fib), fibrin degradation products (FDP) and D-dimer were detected by CA7000 automatic hemagglutination analyzer (Sysmex) and proprietary reagents. The operation process was carried out in strict accordance with the instructions, and the detection was completed within 4h.

Samples were centrifuged for 10 min at 1000 *g* at room temperature and serum was aliquoted and stored at -80°C until use for metabolomic analyses.

### Metabolomic Profiling

The samples were separated by Agilent 1290 Infinity LC ultra-high performance liquid chromatography HILIC column and analyzed by Triple TOF6600 mass spectrometer (AB SCIEX) and Q Exactive series mass spectrometer (Thermo). Positive and negative ion modes of electrospray ionization (ESI) were respectively detected. The original data was converted by ProteoWizard, and then XCMS was used for peak alignment, retention time correction and peak area extraction. Metabolite structure identification and data preprocessing were conducted on the data extracted through XCMS. Subsequently, an assessment of the quality of the experimental data was performed, followed by data analysis. Normalized total peak intensity levels were imported into the ropls R package (version 1.16.0) and the orthogonal partial least squares discriminant analysis (OPLS-DA) algorithm was used for multivariate data analysis, including *R*2 and *Q*2 quality indicators, and the calculation of projected variable importance (VIP) values. VIP reflects variability in pathological stimulus responses explained by specified metabolites.

### Statistical Analysis

IBM SPSS Statistics for Windows software, Version 22.0 (IBM Corp., Armonk, N.Y., USA) was used for statistical analysis. Continuous data of clinical parameters were described as median ± SD and tested by Kolmogorov-Smirnov test to assess the normal distribution of the data. If the data followed a normal distribution, we proceeded to test for homogeneity of variances. If the variances were found to be equal, we conducted independent sample *t*-tests. In cases where the assumption of homogeneity of variances was not met, we performed tests to assess the homogeneity of variances. For data that did not exhibit a normal distribution, we employed the Mann-Whitney *U* test. Frequency variables were recorded as numbers and percentages and compared by χ2 test. In addition, univariate logistic regression was used for statistical analysis of clinical parameters. R (Version 4.3.0) is used for building the machine learning model of random forest (RF) and support vector machine (SVM) to evaluate the importance of variables. R package randomForest, caret and e1071 were used. Venn diagram was plotted by R package VennDiagram.

Receiver operating characteristic (ROC) was plot by R package pROC. It was performed to calculate the sensitivity and specificity of the significant clinical parameters and differential metabolites and investigate the optimal cutoff value for predictions. The optimal cutoff value for sensitivity and specificity was calculated according to the maximal Youden Index. AUROC curves were used to demonstrate the predictive validity. DCA was plotted by R package rmda. By comparing the threshold value to the net benefit, the DCA curve can evaluate the pros and cons of different decision strategies.

## Results

### Metabolomic Profiling

**Fig. 1:**
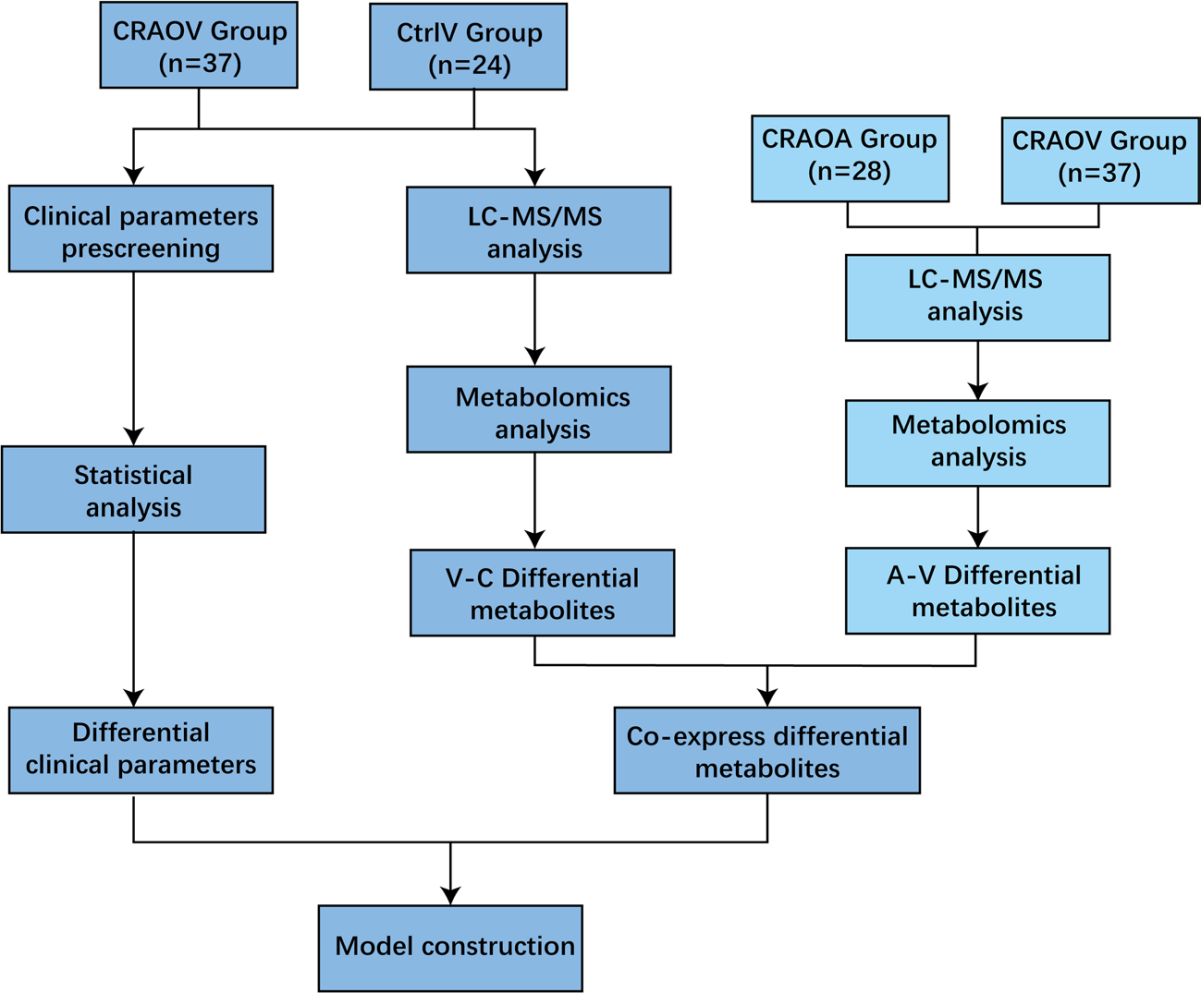
General workflow for this study Clinical parameters of CRAO patients and control patients were screened by statistical methods, and then different clinical parameters was identified. On the other hand, we identified differential metabolites by comparing venous differences between CRAO patients and controls by LC-MS/MS. In addition, we also obtained arteriovenous metabolomics differences between CRAO patients to identify differential metabolites. We then combined the differential metabolites to obtain the co-express differential metabolites. Finally, we combined with the clinical parameters obtained through statistical analysis to build a predictive model and evaluate the model

The overall workflow of the study is shown in Fig. 1. Non-targeted metabolomics analysis was conducted using liquid chromatography-mass spectrometry. Following quality control, data filtering, and normalization, a total of 1259 metabolites were identified through the combination of positive and negative ion models. Among these, 793 metabolites were identified in the positive ion mode, and 466 metabolites were identified in the negative ion mode. All identified metabolites in this study were categorized based on their chemical taxonomy. The lipids and lips-like molecules accounted for the largest proportion, reaching 30.183%. Organic acids and derivatives, as well as organoheterocyclic compounds, also represented a significant proportion of the identified molecular species.

To compare the differences in systemic metabolic profile between CRAO patients and control patients, the venous blood samples from CRAO patients were analyzed and subsequently compared with those from the control patients.

**Fig. 2:**
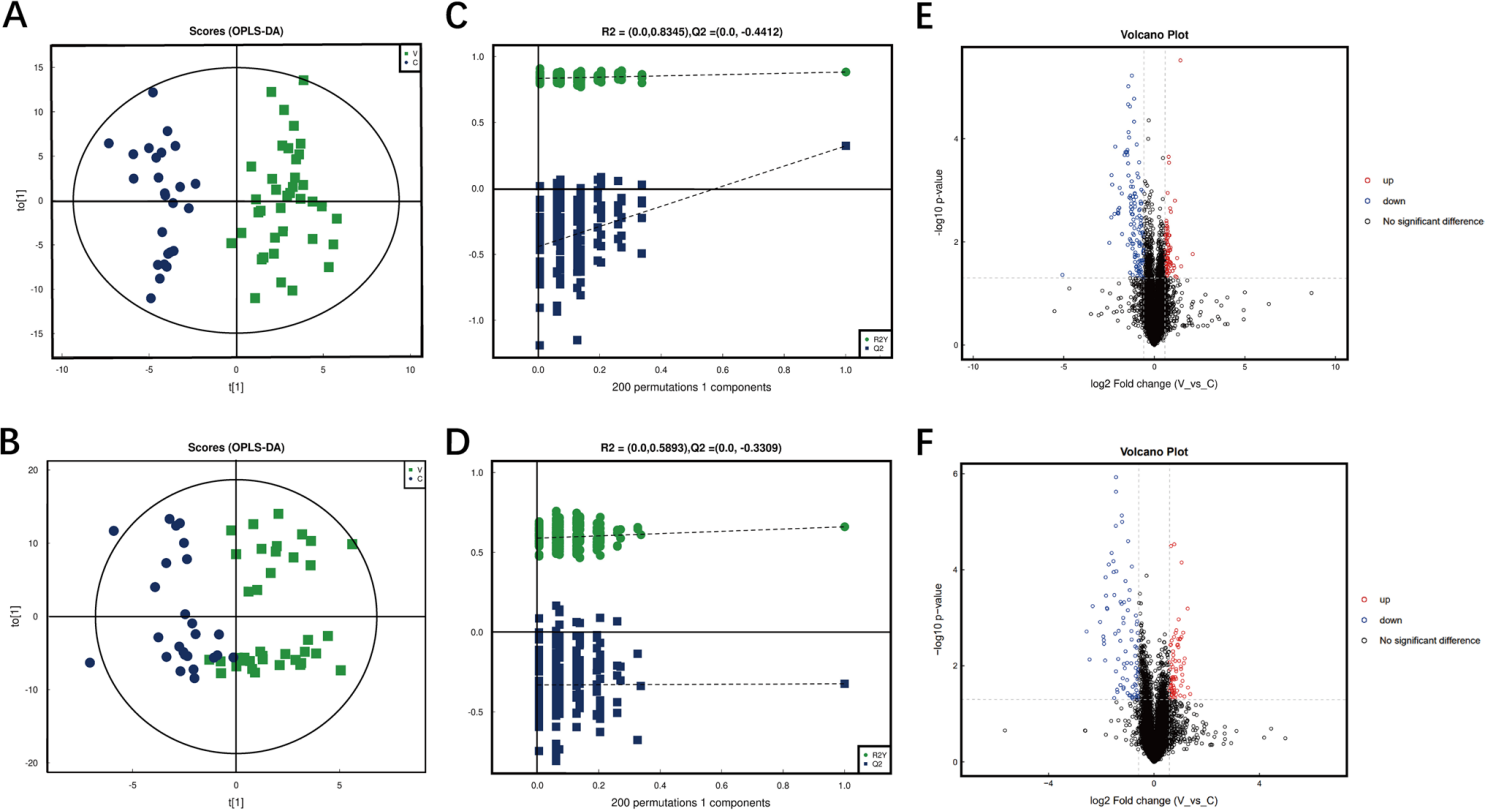
Metabolomics analysis of venous blood differences between CRAO group and control group A, B: Constructing OPLS-DA model for distinguishing CRAO and control group were constructed in negative and positive ion mode. The models show that the two groups can be clearly distinguished; C, D: Permutation test of the models. The model was tested by 200 permutation tests to ensure the validity of the model; E, F: Volcano plot of differential metabolites in negative and positive ion mode. Metabolites with a FC more than 1.5 or less than 0.67, and a *p*-value less than 0.05, were selected as differentially expressed metabolites. Significantly upregulated metabolites are depicted in red, significantly downregulated metabolites are depicted in blue

OPLS-DA was conducted, revealing apparent discrimination among samples (Fig. 2A, B). The supervised classification of OPLS-DA was validated by 200-fold cross-validation. Negative ion model was estimated with a *R*2 value of 0.8345 and a *Q*2 value of 0.4412 at the first component and positive ion model was estimated with a *R*2 value of 0.5893 and a *Q*2 value of 0.3309 (Fig. 2C, D). For the two models, the intercept between *Q*2 regression line and Y-axis is less than 0.05, and with the decrease of replacement retention, *R*2 and *Q*2 decline, and the regression line shows an upward trend, indicating that the model is robust and reliable without overfitting. The criteria for significant differential metabolites involved OPLS-DA VIP>1 and unpaired *t* test with unequal group variance at a threshold of *P*<0.05. Using univariate analysis, we conducted differential analysis on all detected metabolites in both positive and negative ion modes. Metabolites with a fold change (FC) greater than 1.5 or less than 0.67, and a *p*-value less than 0.05, were selected as differentially expressed metabolites. These differentially expressed metabolites were visually presented using volcano plots (Fig. 2E, F).

**Fig. 3:**
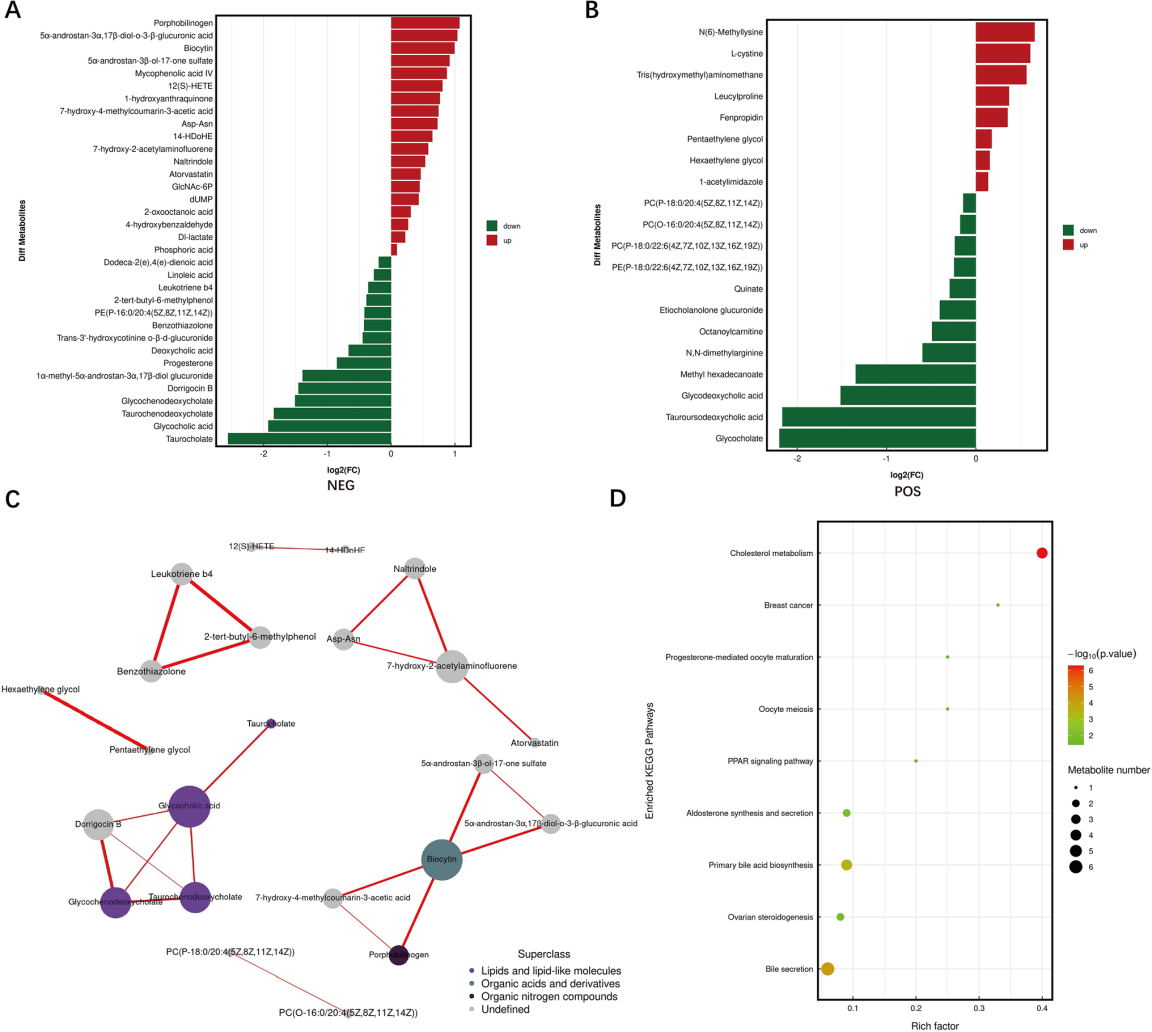
Pathway analysis of venous blood differences between CRAO group and control group. A, B: Differential metabolites between CRAO group and control group in negative and positive ion mode. Red represents upregulated differential metabolites and green represents downregulated differential metabolites. C: Network map of the differential metabolites. D: Pathway enrichment analysis of differential metabolites. Rich factor was the ratio of the number of differentially expressed metabolites located in this pathway to the total number of metabolites located in this pathway among all annotated metabolites

Differential metabolites were screened comprehensively by univariate statistical analysis and multidimensional statistical analysis (Fig. 3A, B). A total of 54 differential metabolites were identified, of which 34 were identified under the anion model and 20 were identified under the anion model. Among these differential metabolites, 27 substances were significantly upregulated, and 27 substances were significantly downregulated in CRAO patients compared to control patients. Through metabolite network mapping, we observed a higher degree of alterations in lipid metabolites among CRAO patients (Fig. 3C). Pathway enrichment analysis revealed significant enrichment of cholesterol metabolism pathway, with different metabolites contributing to a substantial proportion of the overall pathway changes in CRAO patients. Furthermore, the pathway of primary bile acid biosynthesis and bile secretion exhibited considerable alterations in the metabolic profile of CRAO patients (Fig. 3D).

To identify more meaningful metabolites related to CRAO, we compared arterial and venous blood samples from a group of CRAO patients. samples were collected from the site upstream of the obstruction in patients with CRAO who underwent an interventional procedure prior to selective ophthalmic thrombolysis. We hypothesized that the arterial blood collected from the obstruction site might contain differential metabolites that reflect the characteristics of the emboli in comparison to venous blood. Compared with the comparison between venous blood that can reflect the difference in the systemic metabolic status of patients, the comparison between arterial blood and venous blood may be more targeted to reflect the details of embolism. Therefore, we performed metabolomic analysis and comparison of arterial and venous blood sample of CRAO patients.

**Fig. 4:**
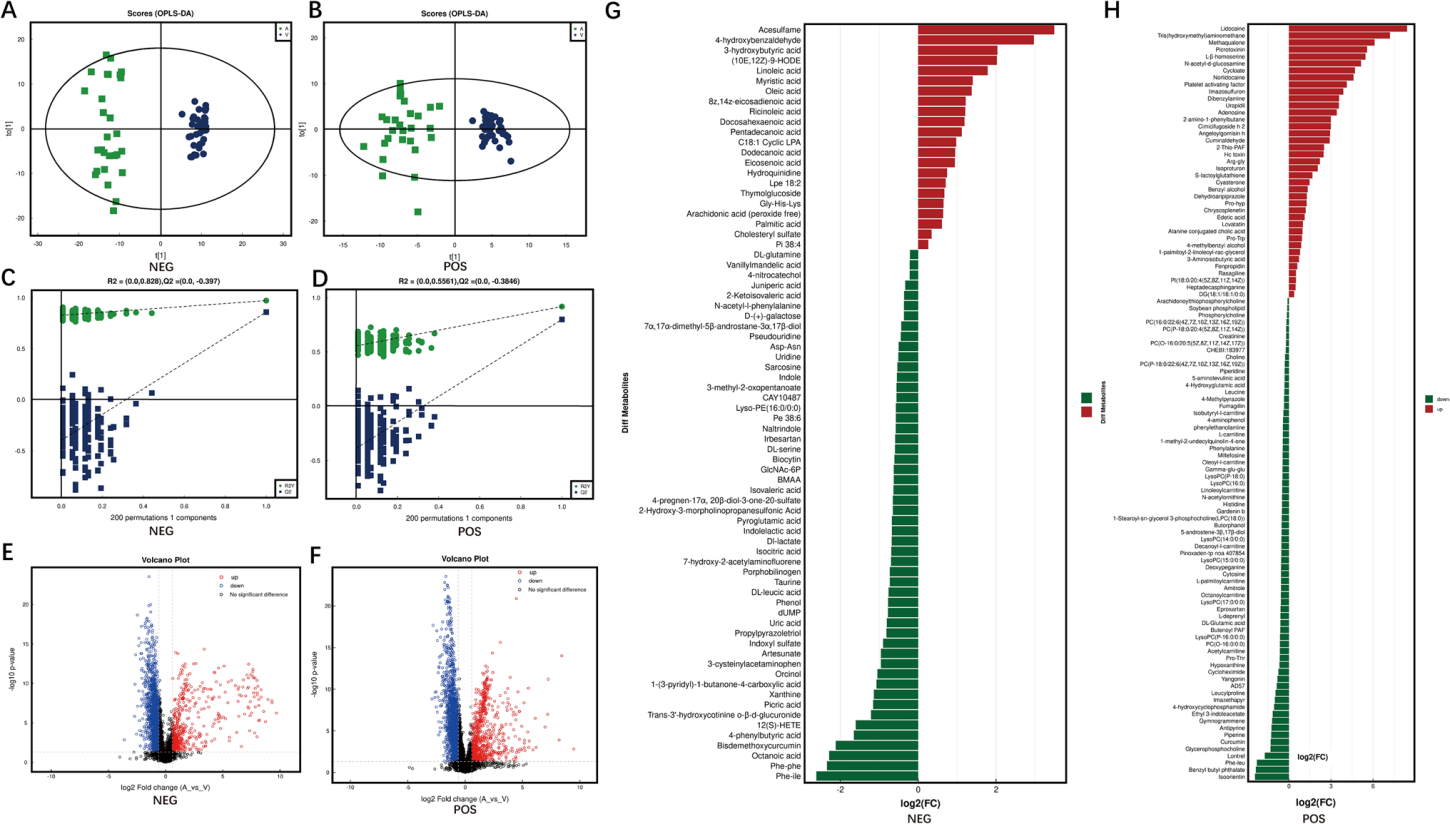
Metabolomics analysis of difference of arterial blood group and venous blood group in CRAO patients. A, B: Constructing OPLS-DA model for distinguishing arterial blood group and venous blood group in negative and positive ion mode. The models show that the two groups can be clearly distinguished; C, D: Permutation test of the models. The model was tested by 200 permutation tests to ensure the validity of the model; E, F: Volcano plot of differential metabolites in negative and positive ion mode. Metabolites with a FC greater than 1.5 or less than 0.67, and a *p*-value less than 0.05, were selected as differentially expressed metabolites. Significantly upregulated metabolites are depicted in red, significantly downregulated metabolites are depicted in blue; G, H: Differential metabolites between arterial blood group and venous blood group in CRAO patients in negative and positive ion mode. Red represents upregulated differential metabolites and green represents downregulated differential metabolites

Similarly, we construct an OPLS-DA model to distinguish sample s and test the model (Fig. 4A, B, C, D). The results of univariate statistical analysis and multidimensional statistical analysis were then used to screen the differential metabolites (Fig. 4E, F). In total, 182 differential metabolites were identified, with 74 identified in positive ion mode and 108 identified in negative ion mode. Among these metabolites, 61 metabolites exhibited an upward trend, and 121 metabolites showed a downward trend (Fig. 4G, H).

**Fig. 5:**
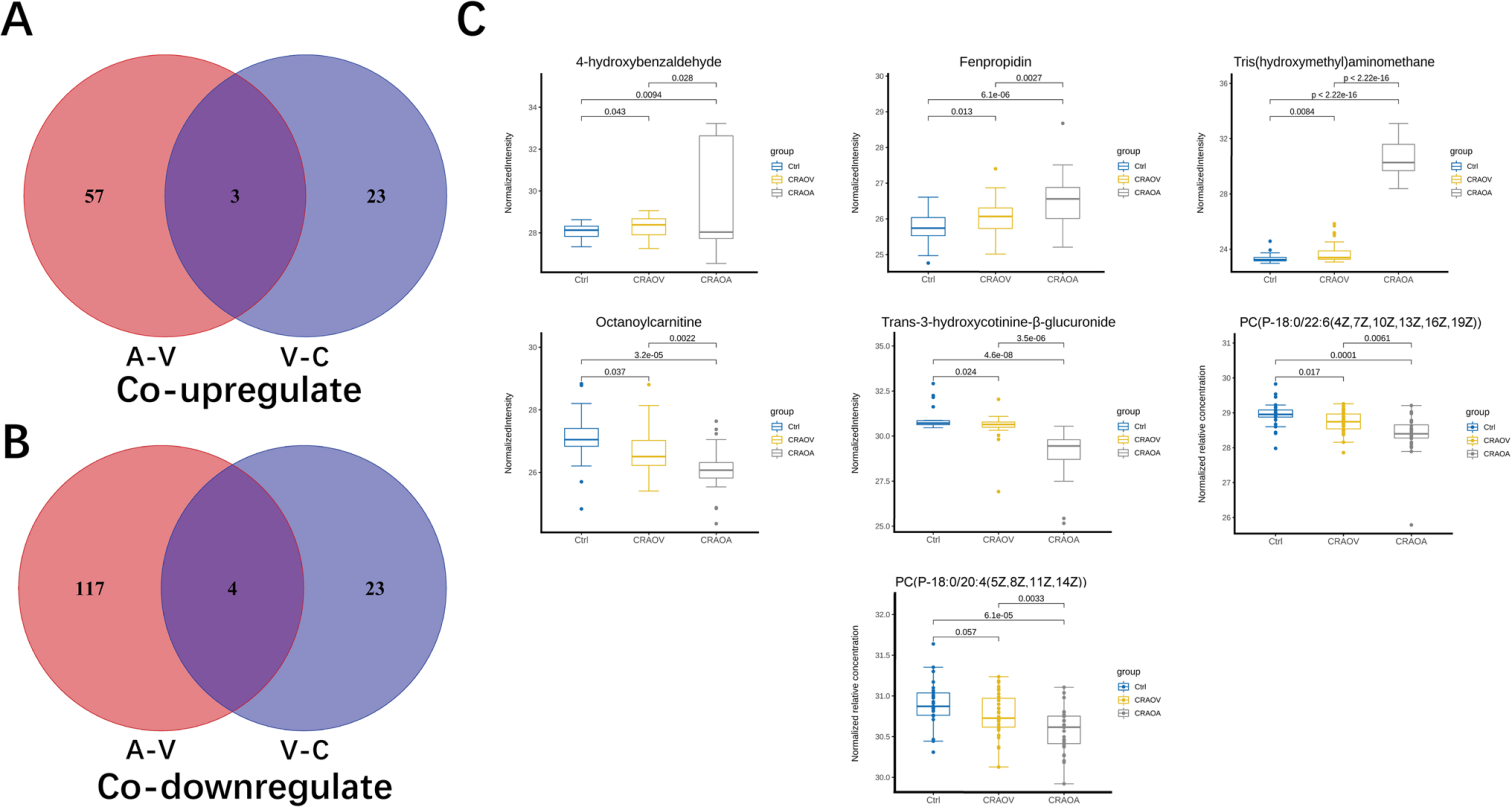
Screening for representative differential metabolites A: Differential metabolites showed an upward trend in the analysis. The red part represents 60 kinds of upregulated differential metabolites comparing the arterial blood of CRAO patients with the venous blood. The blue part represents 26 kinds of upregulated differential metabolites comparing the venous blood of CRAO patients with the venous blood of control patients. The intersection of the Venn diagram represents co-upregulated metabolites among the differential metabolites in the venous blood of CRAO patients compared to the control group and those in the arterial and venous blood of CRAO patients. B: Differential metabolites showed a downward trend in the analysis. The red part represents 121 kinds of downregulated differential metabolites comparing the arterial blood of CRAO patients with the venous blood. The blue part represents 27 kinds of downregulated differential metabolites comparing the venous blood of CRAO patients with the venous blood of control patients. The intersection of the Venn diagram represents co-downregulated metabolites among the differential metabolites in the venous blood of CRAO patients compared to the control group and those in the arterial and venous blood of CRAO patients. C: Box plots represent the relative content of 7 metabolites screened in Fig. 5A and Fig. 5B

To screen for differential metabolites in CRAO patients, we compared venous blood from CRAO patients and control group. We then selected metabolites that displayed the same trend in the comparison of the arterial blood of CRAO patients with the venous blood. Venn diagram represents same trend metabolites among the differential metabolites in the venous blood of CRAO patients compared to the control group and those in the arterial and venous blood of CRAO patients. A total of 7 kinds of substances were identified (Fig. 5A, B). Among the upregulated metabolites, we found tris(hydroxymethyl)aminomethane, fenpropidin, and 4-hydroxybenzaldehyde. In the downregulated category, there were 4 substances: PC(P-18:0/22:6(4Z,7Z,10Z,13Z,16Z,19Z)), PC(P-18:0/20:4(5Z,8Z,11Z,14Z)), octanoylcarnitine, and trans-3-hydroxycotinine-β-glucuronide. The normalized charge-mass ratio, representing relative concentration, is employed to illustrate the concentration gradient of the selected metabolites post-screening (Fig. 5C).

After excluding some non-human metabolites and those metabolites that are clearly not related to CRAO based on prior knowledge, we narrowed down the selection to three metabolites: PC(P-18:0/22:6(4Z,7Z,10Z,13Z,16Z,19Z)), PC(P-18:0/20:4(5Z,8Z,11Z,14Z)) and octanoylcarnitine. We hypothesize that these metabolites could potentially be biomarkers of CRAO.

### Clinical Parameters Analysis

We conducted an analysis of the clinical parameters of the patients from whom blood samples were collected (Table 1). The *p*-value of gender, Hb, HDL-C, ApoA1 and AT-III was less than 0.05, which can be statistically different between the CRAO group and the control group.

**Table 1:** Clinical Characteristics of CRAO patients and control patients

	CRAO (*n* = 37)	Control (*n* = 24)	*P* value
Gender (male)	26(70.27%)	7(29.17%)	0.002
Age (years)	61.08±8.64	65.50±8.10	0.050
Hypertension (%)	18(48.65%)	10(41.67%)	0.593
RBC (×10^12^/L)	4.60±0.57	4.42±0.52	0.251
Hb (g/L)	138.84±15.42	130.21±14.43	0.031
TCh (mmol/L)	4.49±0.92	4.71±1.08	0.404
TG (mmol/L)	1.54(1.22-2.10)	1.72±0.90	0.560
HDL-C (mmol/L)	1.03±0.23	1.25±0.27	0.001
LDL-C (mmol/L)	2.63±0.84	2.71±0.95	0.740
Non-HDL-C (mmol/L)	3.46±0.89	3.45±1.06	0.973
ApoA1 (g/L)	1.22±0.15	1.37±0.15	0.001
ApoB (g/L)	0.87±0.21	0.88±0.25	0.861
ApoA1b (g/L)	1.42(1.20-1.76)	1.62(1.35-2.06)	0.122
PT (s)	10.77±0.67	11.40±2.95	0.756
TT (s)	16.20(15.60-17.15)	15.95(1.5.50-16.55)	0.231
ACT (s)	113.25±12.99	108.51±24.83	0.334
APTT (s)	27.90(26.30-29.10)	27.80(25.45-28.70)	0.521
INR	0.91(0.89-0.97)	0.92(0.88-1.00)	0.652
FIB (g/L)	3.04±0.73	2.89±0.44	0.369
FDP (mg/L)	1.30(1.00-1.80)	1.12(0.81-1.80)	0.595
D-Dimer (mg/L)	0.27(0.23-0.42)	0.32(0.21-0.39)	0.768
AT-III (g/L)	106.49±13.81	94.65±14.09	0.002

*RBC* red blood cell, *Hb* hemoglobin, *TCh* total cholesterol, *TG* triglyceride, *HDL-C* high-density lipoprotein cholesterol, *LDL-C* low-density lipoprotein cholesterol, *Non-HDL-C* non-high-density lipoprotein cholesterol, *ApoA1* apolipoprotein A1, *ApoB* apolipoprotein B, *ApoA1b* apolipoprotein A1b, *PT* prothrombin time, *TT* thrombin time, *ACT* activated coagulation time, *APTT* activated partial thromboplastin time, *INR* international normalized ratio, *FIB* fibrinogen, *FDP* fibrin degradation products, *AT-III* antithrombin-III

To further screen the clinical parameters, univariate logistic regression analysis was conducted on the clinical data. The *p*-value of gender, Hb, HDL-C, ApoA1 and AT-III was less than 0.05, which was considered statistically significant. In terms of the odds ratio (OR) value, gender, HDL-C and Apoa1 are less than 1, indicating a protective effect against CRAO onset. Conversely, the ORs for Hb and AT-III were greater than 1, implying that they might contribute as risk factors for CRAO. However, it is worth noting that the OR values for Gender, Hb, and AT-III were very close to 1, suggesting that their effects might not be substantial. RF and SVM analyzed these parameters and ranked the variables with statistically significant differences as described above. The results are as follows (Table 2).

**Table 2:** Summary of the important clinical parameter for the CRAO patients and non-CRAO patients in various analysis

	Univariate logistic regression		RF	SVM
	Odds Ratio (95%CI)	*P*-Value		
Gender	0.94 (0.88-1)	0.002	Rank 3	Rank 6
Hb	1.04 (1-1.08)	0.040	Rank 9	Rank 9
HDL-C	0.03 (0-0.31)	0.004	Rank 4	Rank 4
ApoA1	0 (0-0.13)	0.003	Rank 1	Rank 2
AT-III	1.07 (1.02-1.12)	0.005	Rank 2	Rank 1

Finally, we evaluated parameters with the OR value of univariate logistic regression as far away from 1 as possible and the *P*-value less than 0.05. Also, RF and SVM were used to rank these clinical parameters. We combined the results of the three methods to screen the variables, and obtained two variables, HDL-C and ApoA1.

### Diagnostic Performance of Combined Model of CRAO

We constructed a clinical model and a combined model of clinical parameters and metabolites for the prediction of CRAO. The clinical model contains two screened clinical parameters. The predictive capacity of the clinical model, as indicated by the AUROC value, was 0.757, reflecting its reasonable ability to discriminate between instances of CRAO and non-CRAO (Fig. 5A).

**Fig. 6:**
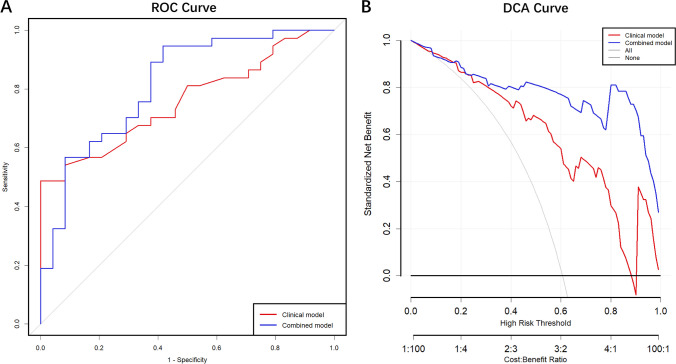
Evaluation of clinical and combined model. A: ROC curves for prediction of CRAO progression based on clinical parameters or a combination of clinical parameters and the differential metabolites in all CRAO and non-CRAO patients. Clinical model include two clinical parameters: HDL-C and ApoA1. Combined model includes two clinical parameters and three differential metabolites: HDL-C, ApoA1, PC (P-18:0/20:4 (5Z,8Z,11Z,14Z), PC (P-18:0/22:6 (4Z,7Z,10Z,13Z,16Z,19Z)) and octanoylcarnitine; B: DCA curve of clinical and combined model

Subsequently, we incorporated the three previously identified significantly differential metabolites and clinical parameters into the logistic regression model. and draw the ROC curve together with the clinical model. We then plotted the ROC curve for this combined model, alongside the clinical model. The results showed that the prediction effect of the model was significantly improved after adding metabolites with the AUROC of 0.815, signifying a significant advancement over the clinical model (Fig. 6A).

To investigate the impact of the combined model on individual patient classification, net reclassification index (NRI) was also calculated. The analysis showed that the combined model outperformed the clinical model in reclassifying non-responders with an NRI of 0.2083. This underscores a meaningful improvement in risk prediction. Additionally, we calculated the integrated discrimination improvement (IDI) to gauge the overall improvement of the model. An IDI value of 0.131 indicated that the new model significantly bolstered its predictive prowess compared to the old model. We also generated a DCA curve to assess the clinical utility of the models. The DCA curve demonstrated that within the High-Risk Threshold range of 0.4 to 1, the combined model consistently exhibited a notably higher net benefit rate compared to the clinical model (Fig 6B). This suggests that the combined model offers enhanced clinical value across this range of risk thresholds.

## Discussion

The aim of this study was to investigate the potential underlying mechanisms of CRAO through the analysis of the metabolome and identifying potential biomarkers. Through screening clinical parameters, we found two blood indexes that may have predictive effects on CRAO occurrence: ApoA1 and HDL-C. And more importantly, by comparing the venous differences between CRAO patients and controls, as well as the arteriovenous differences between CRAO patients, we identified three metabolites: PC (P-18:0/20:4 (5Z,8Z,11Z,14Z)), PC (P-18:0/22:6 (4Z,7Z,10Z,13Z,16Z,19Z)), and octanylcarnitine. We consider these metabolites having the potential to serve as biomarkers of CRAO.

For CRAO, there are currently no suitable biomarkers to predict the onset of CRAO. Current research has focused on markers of inflammation and platelet, such as Neutrophil/Lymphocyte Ratio and platelet distribution width [10, 11, 23]. However, one problem with these indicators is that they can only represent systemic inflammation or abnormal blood clotting. The specificity of such indicators is not high, and their predictive capability for CRAO is not robust. Given that much of the embolus in CRAO patients originates from carotid atherosclerotic plaque, markers of hyperlipidemia serve as better indicators of the systemic condition of patients [24]. Recently, blood lipid indicators, which were widely used in the field of stroke and myocardial infarction, have demonstrated potential in predicting the onset and progression of CRAO [25]. A nationally based cohort study showed a strong association between high HDL-C levels and reduced incidence of CRAO [26]. Also, our study found that HDL-C and ApoA1 can be used as factors to predict the development of CRAO in patients. This can be attributed to the beneficial effects of HDL-C on cardiovascular health [27]. HDL can transfer excess cholesterol from peripheral tissues to the liver in addition, impeding the accumulation of cholesterol in the artery wall and preventing the progression of atherosclerosis [28]. Studies have also shown that the level of HDL-C levels are associated with patient vision outcomes in RAO [29]. ApoA1 is the main component of HDL-C, which also showed a clear correlation with atherosclerosis [30, 31].

Venous blood comparisons between patients and controls may be a good way to explore changes of systemic metabolic characteristics for CRAO [32, 33]. However, better metabolite-specific analyses are required to identify differential metabolites that truly reflect the characteristics of CRAO. During the circulation of blood through organs, the substances it carries can enter cells to participate in metabolism, and the metabolic waste generated enters the bloodstream for removal [34]. Consequently, there can be differences in the levels of metabolites between arterial blood entering organs and venous blood exiting organs, referred to as arteriovenous metabolite differences [35–37]. These differences can reflect the metabolic status of organs, helping understand normal organ homeostasis and potential organ pathologies. Murashige et al. conducted a metabolomic analysis of blood samples taken from the radial artery, coronary sinus, and femoral veins, revealing arteriovenous gradients of circulating metabolites across the heart and leg [38]. They found that the heart mainly consumes fatty acids and secretes glutamine and other nitrogen-rich amino acids. A failing heart consumes more ketones and lactic acid and has a higher rate of proteolysis. Lindeman et.al. analyzed the delayed graft function caused by ischemia-reperfusion injury after kidney transplantation by comparing the differences of arteriovenous metabolites after kidney transplantation [39]. This method has high organ specificity and can reflects the characteristics of local lesion metabolism better than the comparison of venous blood between patients and controls.

For CRAO, alterations in metabolite concentrations upon reaching the embolus site can provide insights into the characteristics of local lesions. Consequently, we analyzed arterial blood near the embolus and compared it with the venous blood of patients. As this analysis inherently encompasses metabolic information for specific organs, we employed Venn diagrams to identify differential metabolites in the venous blood analysis. We propose that these specific metabolites likely play a significant role in the pathogenesis and progression of CRAO.

Studies have shown that the elevated PC(P-18:0/22:6(4Z,7Z,10Z,13Z,16Z,19Z)) and PC(P-18:0/20:4(5Z,8Z,11Z,14Z)) were inversely associated with adiposity indicators and Acceptance and Action Questionnaire for Weight-related difficulties [40].Obesity and overweight have been shown to be important risk factors for CRAO patients [41]. They are also classified as plasmalogens [40]. Plasmalogens have been recognized as crucial constituents of cell membranes and play a significant role in antioxidant processes due to their chemical bonds that are susceptible to attack by reactive oxygen species [42, 43]. In addition, plasmalogens are involved in cholesterol metabolism regulation and transport through a variety of mechanisms [44]. Lipidomics studies have found that plasmalogens is negatively correlated with cardiovascular disease [45]. In addition, the level of plasmalogens in human serum is positively related with serum HDL-C levels and decrease with aging [46].

Our findings revealed that two distinct metabolites, PC(P-18:0/20:4 (5Z,8Z,11Z,14Z)) and PC(P-18:0/22:6 (4Z,7Z,10Z,13Z,16Z,19Z)), exhibited reduced expression levels in CRAO patients compared to control patients. This reduced expression of plasmalogens correlates with the decreased levels of HDL-C. Our results confirm this conclusion. This suggests that plasmalogens may be involved in the pathogenesis of CRAO in patients, possibly through plasmalogens exerting their antioxidant role in atherosclerosis. Concurrently, studies have demonstrated that increased plasmalogen can inhibit cholesterol biosynthesis [47]. The reduced expression of plasmalogens in CRAO patients may result in the accumulation of cholesterol and promote the development of atherosclerosis, which may also be a possible cause of CRAO. Further studies are needed to investigate the potential association between plasmalogens and CRAO.

Octanoylcarnitine is categorized as a kind of medium-chain acylcarnitine which play a role in the β-oxidation of fatty acid [48, 49]. When the rate of β-oxidation exceeds the rate of tricarboxylic acid cycle oxidation, it results in the accumulation of medium-chain acylcarnitine [50]. It is available in dried blood spots for newborn screening to identify those at high risk of medium-chain acyl-CoA dehydrogenase deficiency [51]. Currently, studies based on metabolomics have found that medium-chain acylcarnitine may be related to a variety of diseases, such as cardiovascular and cerebrovascular diseases, insulin resistance and acquired immune deficiency syndrome [52–54].

Improved analytical methods should be considered to explore metabolomic changes in CRAO patients before and after onset. Additionally, we did not classify patients based on embolus type and analyze them separately. The metabolomic characteristics of CRAO patients with different types of emboli may be different, which may have an impact on the analysis. Furthermore, variations in the time from onset to treatment and the evolving metabolic status over time after onset might also impact the outcomes. There is still a difference in the time between the onset of disease and the collection of blood samples, which may cause some metabolites that can reflect the characteristics of the disease to be difficult to identify due to time.

## Abbreviations

CRAO: central retinal artery occlusion
AUROC: area under the receiver operating characteristic curve
RBC: red blood cell
Hb: hemoglobin
TCh: total cholesterol
TG: triglyceride
HDL-C: high-density lipoprotein cholesterol
LDL-C: low-density lipoprotein cholesterol
Non-HDL-C: non-high-density lipoprotein cholesterol
ApoA1: apolipoprotein A1
ApoB: apolipoprotein B
ApoA1b: apolipoprotein A1b
PT: prothrombin time
TT: thrombin time
ACT: activated coagulation time
APTT: activated partial thromboplastin time
INR: international normalized ratio
FIB: fibrinogen
FDP: fibrin degradation products
AT-III: antithrombin-III
ROC: receiver operating characteristic
DCA: decision curve analysis
OR: odds ratio
NRI: net reclassification index
IDI: integrated discrimination improvement
